# P2X4 receptors and neuropathic pain

**DOI:** 10.3389/fncel.2013.00191

**Published:** 2013-10-28

**Authors:** Makoto Tsuda, Takahiro Masuda, Hidetoshi Tozaki-Saitoh, Kazuhide Inoue

**Affiliations:** Department of Molecular and System Pharmacology, Graduate School of Pharmaceutical Sciences, Kyushu UniversityFukuoka, Japan

**Keywords:** P2X4 receptor, microglia, neuropathic pain, spinal cord, fibronectin, CCL21, IRF8, BDNF

## Abstract

Neuropathic pain, a debilitating pain condition, is a common consequence of damage to the nervous system. Neuropathic pain is often resistant to currently available analgesics. A growing body of evidence indicates that spinal microglia react and undergo a series of changes that directly influence the establishment of neuropathic pain states. After nerve injury, P2X4 receptors (P2X4Rs) are upregulated in spinal microglia by several factors at the transcriptional and translational levels. Those include the CC chemokine CCL21 derived from damaged neurons, the extracellular matrix protein fibronectin in the spinal cord, and the transcription factor interferon regulatory factor 8 (IRF8) expressed in microglia. P2X4R expression in microglia is also regulated at the post-translational level by signaling from other cell-surface receptors such as CC chemokine receptor (CCR2). Importantly, inhibiting the function or expression of P2X4Rs and P2X4R-regulating molecules suppresses the aberrant excitability of dorsal horn neurons and neuropathic pain. These findings indicate that P2X4R-positive microglia are a central player in mechanisms for neuropathic pain. Thus, microglial P2X4Rs are a potential target for treating the chronic pain state.

## Introduction

Injury to the nervous system arising from bone compression in cancer, diabetes mellitus, infection, autoimmune diseases, or traumatic injury results in debilitating chronic pain states (so-called neuropathic pain). Characteristic symptoms of neuropathic pain include spontaneous pain, hyperalgesia (increased pain perception of noxious stimuli), and tactile allodynia (pain hypersensitivity to normally innocuous stimuli). Neuropathic pain is refractory to currently available treatments, such as non-steroidal anti-inflammatory drugs and opioids (Costigan et al., 2009). Neuropathic pain is now considered not just a symptom of disease but also the consequence of disordered functioning of the nervous system (Costigan et al., 2009; Beggs et al., 2012).

Extensive lines of evidence from basic pain research using diverse animal models of neuropathic pain indicated that neuropathic pain is a reflection of the aberrant excitability of dorsal horn neurons evoked by peripheral sensory inputs (Woolf and Salter, 2000; Costigan et al., 2009). While neurons have long been considered the only cell type involved in neuropathic pain, recent studies have shown that pathologically altered neurotransmission requires communication with glial cells, in particular microglia, activated in the spinal cord in response to peripheral nerve injury (PNI; Watkins et al., 2001; Tsuda et al., 2005; McMahon and Malcangio, 2009; Ren and Dubner, 2010; Tsuda et al., 2013).

Microglial cells are resident macrophages in the central nervous system (CNS), which derive from primitive macrophages in the yolk sac (Ginhoux et al., 2010). In the adult, microglia are ubiquitously distributed throughout the brain and spinal cord and have small cell body bearing branched and motile processes, which monitor the local environment in the CNS (Davalos et al., 2005; Nimmerjahn et al., 2005). Microglia show a stereotypical long-term response to a wide range of stimuli that threaten physiological homeostasis, including PNI. In response to PNI, microglia activation** in the spinal cord progresses through hypertrophic morphology, an increase in cell number, and altered gene expression (Tsuda et al., 2005; Suter et al., 2007; Tsuda et al., 2009b). Activated microglia induce or enhance expression of various genes including neurotransmitter receptors such as purinergic P2 receptors (Pocock and Kettenmann, 2007). By responding to extracellular stimuli such as ATP, activated glia evoke various cellular responses such as production and release of bioactive factors including cytokines and neurotrophic factors (Inoue, 2006), which in turn leads to hyperexcitability of dorsal horn neurons and neuropathic pain.

Among purinergic P2 receptors [ionotropic receptors (P2XRs) and metabotropic receptors (P2YRs)], activated microglia express several subtypes of P2XRs and P2YRs, and these receptors play a key role in establishing and maintaining neuropathic pain states (Tsuda et al., 2012, 2013). In this article, we highlight recent advances that further increase our understanding of the mechanisms underlying neuropathic pain, with a specific focus on P2X4 receptor (P2X4R) in spinal microglia after PNI.

### P2X4R in spinal microglia and neuropathic pain

**Figure 1:**
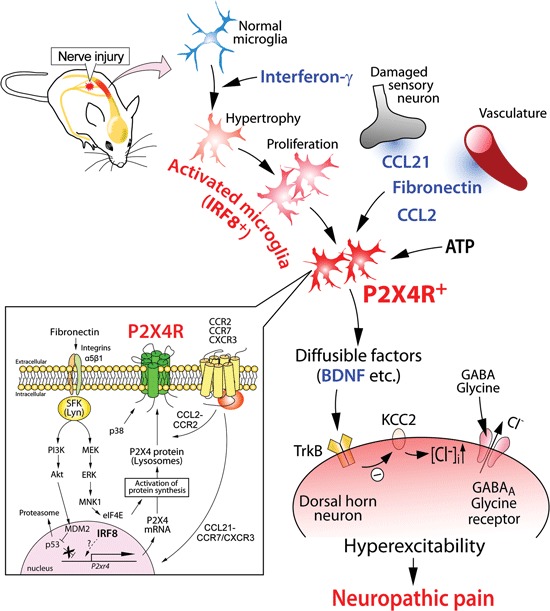
**Schematic illustration of the potential mechanisms by which P2X4R in activated microglia modulate pain signaling in the dorsal horn after PNI**. Nerve injury activates microglia in the dorsal horn of the spinal cord. Activated microglia show increased expression of P2X4R. The upregulation of microglial P2X4R expression involves signaling by fibronectin and chemokine (C-C motif) ligand 21 (CCL21). CCL2 signaling promotes P2X4R trafficking to cell surface of microglia. P2X4R is activated by ATP and, in turn, release bioactive diffusible factors, such as BDNF. BDNF downregulates the potassium-chloride transporter KCC2 via TrkB, causes an increase in intracellular [Cl^-^], and leads to the collapse of the transmembrane anion gradient in dorsal horn neurons which in turn induces depolarization of these neurons following stimulation by GABA and glycine. The resultant hyperexcitability in the dorsal horn pain network induced by factors from activated microglia may be responsible for neuropathic pain.

The first observation that demonstrated the important role of P2X4R in neuropathic pain was that established tactile allodynia after PNI was reversed by pharmacological blockade of P2X4Rs in the spinal cord (Tsuda et al., 2003). Immunohistochemical studies revealed that expression of P2X4Rs in the spinal cord was upregulated exclusively in microglia. These results indicated that PNI-induced pain hypersensitivity depended on ongoing signaling via microglial P2X4Rs. Furthermore, animals with P2X4R knock-down or knock-out in the spinal cord were resistant to PNI-induced tactile allodynia (Tsuda et al., 2003; Ulmann et al., 2008; Tsuda et al., 2009a), indicating a necessity for P2X4Rs. The impact of microglial P2X4R stimulation in transforming tactile information to pain was demonstrated by an *in vivo* microglia transfer approach (Tsuda et al., 2003). It was found that intrathecal delivery of P2X4R-stimulated microglia caused normal rats to develop allodynia and indicated that microglial P2X4R stimulation is sufficient (Tsuda et al., 2003, 2005). Furthermore, it was demonstrated that activation of microglial P2X4Rs stimulated the synthesis and release of brain-derived neurotrophic factor (BDNF; Ulmann et al., 2008; Trang et al., 2009) and that BDNF then causes an altered transmembrane anion gradient in a subpopulation of dorsal horn lamina I neurons presumably through the downregulation of the neuronal chloride transporter KCC2, which in turn renders GABA and glycine effects depolarizing, rather than hyperpolarizing, in these neurons (Coull et al., 2005; Figure 1). Thus, P2X4R-stimulated microglia release BDNF as a crucial factor to signal lamina I neurons, causing an aberrant nociceptive output that contributes to neuropathic pain (Beggs et al., 2012). Therefore, microglial P2X4Rs are central players in the pathogenesis of neuropathic pain.

### Regulation of P2X4R expression in microglia: transcriptional, translational, and post-translational levels

Upregulation of P2X4R expression in microglia is a key process in the pathogenesis of neuropathic pain. Several studies have identified molecules involved in the upregulation of P2X4R expression in spinal microglia after PNI.

A clue to identifying an inducer of P2X4R expression in microglia was that microglial P2X4R upregulation was observed following PNI but not peripheral tissue inflammation (Tsuda et al., 2003), raising the possibility that a factor derived from injured primary afferent sensory neurons might be involved. Recently, it was shown that the chemokine CCL21 [chemokine (C-C motif) ligand 21] was induced in injured dorsal root ganglion (DRG) neurons and transported to the central terminals of the dorsal horn (Biber et al., 2011). Mice treated with CCL21-neutralizing antibody and mice deficient for CCL21 showed attenuation of tactile allodynia and microglial P2X4R upregulation. CCL21 treatment increased the expression of P2X4R in cultured microglia, indicating a direct action of CCL21 on microglia. Intrathecal supply of CCL21 in CCL21-deficient mice rescued PNI-induced tactile allodynia in those mice. Thus, CCL21 derived from injured DRG neurons directly contributes to microglial P2X4R expression and neuropathic pain (Biber et al., 2011; Figure 1).

Because blood–spinal cord barrier functions collapse after PNI occurs (Beggs et al., 2010; Echeverry et al., 2011), proteins leaking from the blood might change P2X4R expression in microglia. The extracellular matrix protein fibronectin might be such a protein. The level of fibronectin protein was elevated in the dorsal horn after PNI (Nasu-Tada et al., 2006; Echeverry et al., 2011). Fibronectin stimulation induces upregulation of P2X4R mRNA and protein in primary cultured microglial cells (Nasu-Tada et al., 2006). Using integrin blockers *in vitro* and *in vivo*, it was shown that fibronectin/integrin signaling was crucial for augmentation of P2X4R expression and PNI-induced tactile allodynia (Tsuda et al., 2008a). Furthermore, intrathecal injection of fibronectin to naïve animals produced tactile allodynia, a behavior not observed in P2X4R-deficient mice administered fibronectin (Tsuda et al., 2008a). Regarding intracellular signaling mechanisms underlying P2X4R upregulation by fibronectin, microglial Lyn tyrosine kinase, a member of Src-family kinases (SFKs) that belong to the non-receptor protein tyrosine kinase family, is an important molecule as upregulation of P2X4R gene expression in response to fibronectin is not observed in microglial cells lacking Lyn (Tsuda et al., 2008b). In spinal cord microglial cells, Lyn was the predominant SFK (Tsuda et al., 2008b) amongst the five members (Src, Fyn, Lck, Yes, and Lyn) that are known to be expressed in the CNS (Salter and Kalia, 2004). Lyn expression in the spinal cord *in vivo* is highly restricted to microglia, and following PNI the level of Lyn is increased (Tsuda et al., 2008b), which is interferon-γ signaling dependent (Tsuda et al., 2009b). Mice lacking Lyn suppress PNI-induced tactile allodynia and the upregulation of spinal P2X4R expression after PNI (Tsuda et al., 2008b). Following Lyn tyrosine kinase activation, two intracellular signaling cascades are distinctly activated: one is a pathway through phosphatidylinositol 3-kinase (PI3K)-Akt and the other is through mitogen-activated protein kinase kinase (MAPK kinase, MEK)-extracellular signal-regulated kinase (ERK; Tsuda et al., 2009c). Signaling through the PI3K-Akt pathway induced degradation of p53 via mouse double minute 2 in a proteasome-dependent manner. The consequence of an attenuated repressive effect of p53 may be associated with enhanced P2X4 gene expression. However, activated MEK-ERK signaling in microglia exposed to fibronectin enhanced eukaryotic translation initiation factor 4E (eIF4E) phosphorylation status via activated MAPK-interacting protein kinase-1, which may play a role in regulating P2X4 expression at translational levels (Figure 1). The Lyn-ERK signaling pathway seems likely to be active in spinal microglia after PNI as pharmacological inhibition of SFK effectively suppressed ERK activity in spinal microglia (Katsura et al., 2006). Thus, Lyn might be a key kinase in the molecular machinery mediating the upregulation of P2X4R in microglia (Figure 1).

To detect extracellular ATP, P2X4Rs are expressed on the cell surface of microglia. However, a large amount of P2X4R protein within microglia (and macrophages) localizes predominantly to intracellular lysosomal compartments (Qureshi et al., 2007). Interestingly, P2X4R protein remains stable within the proteolytic environment of lysosomes. How P2X4R protein is recruited to the cell surface of microglia remains elusive, but recent studies have shown that trafficking of P2X4R protein to the cell surface occurs when microglia are stimulated by a Toll-like receptor (TLR)4 agonist lipopolysaccharide (Boumechache et al., 2009; Toulme et al., 2010) or the Ca^2+^ ionophore, ionomycin (Qureshi et al., 2007). Furthermore, the chemokine CCL2 increased P2X4R protein levels on the cell surface (without changing total cellular expression) via CC chemokine receptor (CCR2) (Toyomitsu et al., 2012). Notably, CCL2 changed the distribution of lysosomes with P2X4R protein within microglial cells and induced the release of a lysosomal enzyme (Toyomitsu et al., 2012). Thus, CCL2 might promote the expression of P2X4R protein on the cell surface of microglia through exocytosis of P2X4R-containing lysosomes (Figure 1). A recent study using single-molecule imaging to track P2X4Rs in the processes of microglia showed that lateral mobility of P2X4Rs is enhanced in activated microglia by the p38 MAPK pathway that selectively regulates slowly mobile P2X4Rs (Toulme and Khakh, 2012). These results indicated that microglial P2X4Rs are dynamically regulated on the cell surface (Figure 1). Thus, post-translational regulation to enhance P2X4R expression and mobility on the cell surfaces of microglia might render cells hyper-responsive to extracellular ATP, which may be important in neuropathic pain.

### Transcriptional factor for reactive states of microglia

PNI activates microglia and converts them to reactive phenotypes through the activation of gene transcription. For example, as described above, activated microglia with high levels of P2X4R expression are essential for PNI-induced neuropathic pain. Appropriate changes in gene expression patterns required for transformation into reactive phenotypes might be tightly regulated by cell type-specific transcription factors. Recent studies identified interferon regulatory factor 8 (IRF8) as a transcription factor in microglia (Horiuchi et al., 2012; Masuda et al., 2012; Minten et al., 2012; Kierdorf et al., 2013) that was critical for their activation and neuropathic pain (Masuda et al., 2012). IRF8 is a member of the IRF family (IRF1–9), and is expressed in immune cells such as lymphocytes and dendritic cells (Tamura et al., 2008). Within the spinal cord, IRF8 expression was markedly upregulated in microglia, but not in neurons or astrocytes, after PNI (Masuda et al., 2012). The microglia-specific upregulation of IRF8 occurs as early as postoperative day 1, peaks on day 3, and persists for at least several weeks. IRF8-deficient mice showed a reduction of PNI-induced tactile allodynia with no change in basal mechanical sensitivity. Notably, suppressing the upregulated expression of spinal IRF8 after PNI by intrathecal administration of a small interfering RNA (siRNA) targeting IRF8 in wild-type mice with allodynia caused a significant recovery of tactile allodynia. This indicated an ongoing activation of IRF8 in spinal microglia. *In vitro* and *in vivo* studies demonstrated that IRF8 promoted the transcription of P2X4R and other molecules associated with reactive phenotypes [innate immune response (TLR2), chemotaxis (P2Y12R and the chemokine receptor CX3CR1), and inflammatory components (interleukin-1*β*, cathepsin S, and BDNF)]. Nevertheless, nerve injury-induced proliferation of spinal microglia was not affected by IRF8 deficiency, indicating IRF8 deficiency does not impair all reactive processes of microglia. Rather the transcription factor contributes to determining the reactive phenotypes of microglia by changing the expression of a set of genes including P2X4R (Figure 1). However, several important issues remain. How is IRF8 expression induced in microglia after PNI? To determine whether IRF8 directly binds to promoter regions of these genes is also important. Answers to these issues will provide new insights into the molecular mechanisms underlying microglial activation and neuropathic pain.

### Markers of activated microglia *in vivo*

A widely used index for predicting the status of microglia activity *in vivo* is the expression levels of microglial markers [complement receptor 3 (CR3, recognized by OX-42) and Iba1]. Increased immunolabeling of these markers is a principal feature of a variety of PNI models (Tsuda et al., 2005; Suter et al., 2007), but it remains unclear whether their expression can be linked to tactile allodynia following PNI as in some cases CR3 expression is not well correlated with the degree of allodynia (Colburn et al., 1997; Winkelstein and DeLeo, 2002; Tsuda et al., 2005; Suter et al., 2007). In addition, OX-42 immunofluorescence was still observed in P2X4R-knockdown animals that had attenuated PNI-induced allodynia (Tsuda et al., 2003). In contrast, as described above, microglia-specific molecules including P2X4R and IRF8 are necessary for the pathology of neuropathic pain, in both its development and maintenance phases. Therefore, alterations in the expression or phosphorylation of these molecules are likely to be more useful markers to assess reactive states of microglia *in vivo*.

## Concluding remarks

We have primarily focused on the role of P2X4R expressed in spinal microglia in neuropathic pain (Figure 1). Pharmacological, molecular, and genetic studies on P2X4Rs described above provide compelling evidence that P2X4R-positive microglia are a central player in the mechanisms of neuropathic pain and might be promising targets for treating neuropathic pain. Furthermore, the upregulation of microglial P2X4R expression has also been reported in animal models of stroke (Cavaliere et al., 2003), brain tumor (Guo et al., 2004), traumatic brain injury (Guo and Schluesener, 2005; Zhang et al., 2007), spinal cord injury (Schwab et al., 2005), epilepsy (Ulmann et al., 2013), and in human acute inflammatory demyelinating polyradiculoneuropathy (Zhang et al., 2008). Recently, it was demonstrated that P2X4R-positive microglia are essential for morphine-induced hyperalgesia via a P2X4R-BDNF-KCC2 disinhibition cascade between microglia and dorsal horn neurons (Ferrini et al., 2013). It is expected that an increased understanding of the phenotype of P2X4R-positive microglia will provide us with exciting insights into pain mechanisms and clues to aid the development of new therapeutic agents for the management of neuropathic pain and other CNS diseases.

## Conflict of interest statement

The authors declare that the research was conducted in the absence of any commercial or financial relationships that could be construed as a potential conflict of interest.
